# Psychosocial and Mental Health Determinants of Suicidal Behavior Among Nursing Students: A Cross-Sectional Study in Mexico

**DOI:** 10.3390/nursrep15120441

**Published:** 2025-12-10

**Authors:** Margarita L. Martinez-Fierro, Leticia A. Ramirez-Hernandez, Perla M. Trejo-Ortiz, Georgina Lozano-Razo, Javier Zavala-Rayas, Sodel Vazquez-Reyes, Perla Velasco-Elizondo, Alejandro Mauricio-Gonzalez, Roxana Araujo-Espino, Fabiana E. Mollinedo-Montaño, Jose R. Gutierrez-Camacho, Idalia Garza-Veloz

**Affiliations:** 1 Doctorado en Ciencias con Orientación en Medicina Molecular, Unidad Academica de Medicina Humana y Ciencias de la Salud, Universidad Autonoma de Zacatecas, Carretera Zacatecas-Guadalajara Km.6, Ejido la Escondida, Zacatecas 98160, Mexico; margaritamf@uaz.edu.mx (M.L.M.-F.); perlatrejo@uaz.edu.mx (P.M.T.-O.); vazquezs@uaz.edu.mx (S.V.-R.); pvelasco@uaz.edu.mx (P.V.-E.); amgdark@uaz.edu.mx (A.M.-G.); roxana.araujo@uaz.edu.mx (R.A.-E.); fabiana.mollinedo@uaz.edu.mx (F.E.M.-M.); rob_gutierrez_mm@uaz.edu.mx (J.R.G.-C.); 2 Psychology Program, Intercontinental University, Insurgentes Sur, Calz. de Tlalpan 4303, Sta Ursula Xitla, Ciudad de México 14420, Mexico; 3 Unidad Academica de Matematicas, Universidad Autonoma de Zacatecas, Zacatecas 98160, Mexico; 4 Unidad Academica de Psicologia, Universidad Autonoma de Zacatecas, Zacatecas 98160, Mexico; glozraz@uaz.edu.mx (G.L.-R.); jzavala@uaz.edu.mx (J.Z.-R.)

**Keywords:** suicide, suicidal behavior, hopelessness, depression, nursing students

## Abstract

**Background:** Nursing students face emotional and psychological challenges stemming from early clinical exposure, intense academic pressure, and persistent social stigmas. These stressors can contribute to mental health deterioration and increase the risk of suicidal thoughts and behavior. **Objective:** To evaluate the psychosocial context and identify risk and protective factors contributing to suicidal behavior in undergraduate nursing students. **Methods:** This cross-sectional study included 433 undergraduate nursing students and utilized validated psychological instruments to assess suicidal behavior, emotional distress, impulsivity, anhedonia, mental health, and perceived social support. Data were analyzed using descriptive statistics, bivariate tests, exploratory factor analysis, and multivariate modeling to identify key predictors of suicidal behavior. Network visualization was used to integrate significant point-biserial correlations with factor loadings. **Results:** Among 433 nursing students (77.8% women, 93.8% cisgender, mean age 19), 15.2% showed clinically significant suicidal risk. Suicidal behavior was more frequent among women and students living away from home (*p* < 0.05). Higher levels of impulsivity, ADHD symptoms, and especially moderate-to-severe hopelessness (*p* < 0.001) were strongly associated. Hazardous alcohol use was also a significant risk factor (*p* < 0.01), while strong material and emotional support showed a protective effect (*p* < 0.05). Two psychological dimensions, emotional distress/impulsivity and hopelessness/low support, explained most of the variance. **Conclusions:** 1 in 7 nursing students show clinically relevant suicidal risk, particularly those with heightened hopelessness, emotional dysregulation or hazardous alcohol use. Protective social support plays a key mitigating role. These results underline the urgent need for tailored mental health interventions that specifically address emotional regulation and hopelessness, while reinforcing social support systems within nursing education contexts.

## 1. Introduction

Suicidal behavior, which includes suicidal thoughts, self-harm, suicide and suicide attempts, represents a major global public health concern [1]. According to the World Health Organization (WHO), it is the third leading cause of death among individuals aged 15 to 29 [2]. This represents a substantial burden in terms of potential years of life lost and disability-adjusted life years [3]. Each year, approximately 727,000 people die by suicide worldwide [2]. The economic impact is also significant, as the direct and indirect costs associated with mental health disorders exceed 4% of the gross domestic product in many countries, according to the Organization for Economic Cooperation and Development (OECD) [4]. Among university-aged individuals, suicide is the second leading cause of death globally [5].

Suicidal behavior stems from a complex interplay of psychological, social, biological, cultural and environmental factors [6]. Identified risk factors range from mental health conditions such as depression and anxiety to adverse socioeconomic circumstances, trauma and access to lethal means [7]. From a neurobiological perspective, dysregulation of neurotransmitters, particularly serotonin, has been associated with suicidal behaviors [8]. Young adults, particularly those in university settings, experience unique stressors, such as academic pressure, evolving social roles and future expectations, that can exacerbate vulnerabilities to mental health challenges [9]. For instance, during the critical transition into professional studies such as medicine or nursing, emotional exhaustion and limited time for self-care can increase their risk [10]. It has also been reported that nursing training, which involves clinical practice (along with theoretical and didactic teaching), can expose students to stressors that students in many other disciplines do not experience [11,12]. These stressors may include traumatic events and interpersonal challenges with staff [13]. The COVID-19 pandemic caused by SARS-CoV-2, which led to the incorporation of many students worldwide into practice, introduced additional stressors, such as isolation from family and friends [14] and high prevalence of depression, anxiety, and post-traumatic stress disorder (PTSD) [15].

Concerns about suicide risk in nursing students have been documented for many years [16,17]. Suicidal behavior in this population appears multifactorial and has been associated with mental and physical health problems, substance use, work-related stress, and interpersonal difficulties [18,19]. The temporal onset of this vulnerability remains unclear, and although precise suicide rates remain unknown, nursing students consistently report higher levels of anxiety and depression compared to peers in other academic programs [12]. Individuals with pre-existing traits associated with suicide risk may be more likely to enter the nursing profession, and additional stressors inherent to clinical practice may further intensify this susceptibility [20,21]. Recent evidence from a nationwide Swedish cohort showed that nursing students have a higher risk of suicide compared to education students, particularly within the first three years of training [22]. This elevated risk was partly explained by higher rates of mental illness and prior self-harm; however, the persistence of the association after adjustment suggests that unmeasured vulnerabilities (such as personality traits or family history) may also contribute [22]. Despite not all individuals with mental illness or self-harm seeking healthcare services, making the true prevalence difficult to estimate [19], several reports consistently corroborate the increased risk of suicide among nurses and nursing students [23,24,25].

Psychological factors such as self-esteem, impulsivity, depression, life stress, and perceived social support play an important role in suicidal ideation among nursing students. Low self-esteem, closely linked to suicidal thoughts and attempts in high-stress environments, compromises identity formation and adaptability to change [26]. Individuals with a history of suicidal ideation typically exhibit lower self-esteem than those without such experiences [26]. Depression and life stress are major contributors to suicidal ideation in university populations, whereas high self-esteem acts as a protective factor, including among nursing students [27]. Impulsivity, which facilitates the progression from suicidal ideation to action, is strongly associated with suicidal behaviors in university populations and may be amplified in nursing students due to academic and emotional pressures [28,29,30]. In the same sense, hopelessness in nursing students has been associated with anxiety, insomnia, and feelings of inadequacy, factors exacerbated by academic and clinical demands, contributing to increased emotional vulnerability [31,32]. Attention-deficit/hyperactivity disorder (ADHD) symptoms are linked to greater suicidal thoughts and behaviors in young adults, even after accounting for depression and anxiety [33]. In nursing students, disorganization and inattention may contribute to poor academic performance, stress, and frustration, increasing susceptibility to psychological distress, while impulsivity inherent to ADHD may promote self-destructive decision-making [34].

According to the above, nursing students face high levels of academic stress, early exposure to human suffering, and a substantial emotional burden during clinical training, making them a particularly vulnerable population for mental disorders and/or suicidal behavior development [35]. Despite extensive research, the complexity of suicidal behavior means that common risk factors remain elusive, emphasizing the need for situation-specific studies by academic profiles. In this context, our current study aims to identify the psychosocial determinants associated with suicidal behavior among nursing students. Adopting a multidimensional approach will enable us to provide essential insights to inform the development of targeted preventive strategies and interventions for this vulnerable group, thereby helping to reduce the incidence of suicide and improve mental health outcomes.

## 2. Materials and Methods

### 2.1. Study Design and Participants

This cross-sectional study was conducted in Zacatecas, Mexico, between November and December 2023. The study population consisted of undergraduate students enrolled in the Bachelor of Nursing program at the Autonomous University of Zacatecas “Francisco García Salinas”. The Autonomous University of Zacatecas is an internationally recognized public institution with programs accredited by international organizations. In particular, the Nursing program has a long history of training health professionals in the state of Zacatecas. This school is the oldest in nursing education and has two current national re-accreditations (Interinstitutional Committees for the Evaluation of Higher Education: CIEES; and Mexican Council for Nursing Accreditation: COMACE), as well as two international accreditations from NOM-ISO 9001:2025 and the Accreditation Agency of Santiago, Chile. It also has a Favorable Academic Technical Opinion (OTA 2025-30), which allows students to enter clinical fields within public and private health institutions in Mexico. Eligible participants were officially recruited at the Academic Unit of Nursing (with authorization and approval from the academic authorities), regardless of sex, and were aged between 18 and 35. They had to provide written informed consent to participate in the study. Students were excluded if they did not complete the psychometric instruments, withdrew from the study, or if more than 80% of the required data was missing.

### 2.2. Sample and Sampling

A stratified random sampling strategy with proportional allocation was employed. The strata were defined according to contextual variables previously associated with suicidal behavior, including sex, local or non-local status (i.e., from another town), campus and semester of enrolment. The reference population comprised around 1863 students enrolled in the Nursing program (based on official university enrolment reports for the 2023–2024 academic cycle). Estimates for mental health indicators in Mexican youth were used to calculate the sample size: suicidal ideation (6.9%; ENSANUT [36]), depression (15.4–19.5%), anxiety (19.3–31.3%; ENBIARE [37]) and ADHD (8.8%; WHO [38]). With a maximum expected error of 5% and a 95% confidence level, the minimum required sample size was determined to be 429 students. Based on these prior national surveys of Mexican youth and using Cochran’s formula for a single proportion, we determined that a minimum of 429 nursing students would be required to achieve 95% confidence with a 5% margin of error. We therefore aimed to recruit around 433 participants to ensure adequate statistical power. Sample allocation across strata was based on official enrolment data, and proportional calculations were performed using Microsoft Excel^®^ 2021 (Microsoft Corp., Redmond, WA, USA).

### 2.3. Techniques and Instruments for Data Collection

Data were collected via an online, self-administered survey that incorporated a comprehensive set of validated questionnaires designed to evaluate risk and protective factors associated with suicidal behavior. The following instruments were employed: The Beck Scale for Suicidal Ideation; the Beck Hopelessness Scale; the Depression, Anxiety and Stress Scale (DASS-21); Plutchik’s Impulsivity Scale; the International Physical Activity Questionnaire (IPAQ); the Adult ADHD Self-Report Scale (ASRS v1.1), the Medical Outcomes Study Social Support Survey (MOS), the Alcohol Use Disorders Identification Test (AUDIT), the Self-Injury Questionnaire (SIQ), the Cannabis Addiction Test (CAST), the Fagerström Test for Nicotine Dependence (FTND), the Alcohol, Smoking and Substance Involvement Screening Test (ASSIST, Cocaine Section) and the Drug Use Situations Inventory (DUSI). The Beck Scale for Suicidal Ideation (21 items) assessed the presence and severity of suicidal thoughts; the Beck Hopelessness Scale (20 items) measured negative expectations for the future; the Plutchik’s Impulsivity Scale (15 items) assessed the degree of behavioral control; the MOS measured social support in four dimensions (material, emotional, affective, and creative); and the Adult ADHD Self-Report Scale (ASRS v1.1) identified symptoms compatible with ADHD in young adults. All instruments used have versions that have been validated in Mexican or broader Hispanic populations, ensuring adequate validity and reliability for use in this context [39,40,41,42,43,44,45,46,47,48,49,50,51,52,53] (see Table S1 in Supplementary File S1, for additional information regarding reliability and validity of psychological and substance use instruments used in the study). Anhedonia was evaluated using a single item developed in accordance with the DSM-5 definition, which describes anhedonia as a markedly diminished interest or pleasure in previously enjoyable activities [54]. Participants were asked: “During the past six months, have you felt less motivated or experienced reduced pleasure in activities that previously caused you enjoyment?” Response options ranged from “always,” “almost always,” “sometimes,” “almost never,” to “never.” This single-item approach was used to capture the core subjective component of anhedonia in non-clinical samples.

The term “foreigner” in this study refers to students who relocated from another town or municipality within the country to attend the nursing program, rather than international students. Local students were those residing in the same city where the university is located.

### 2.4. Ethical Considerations

The study was conducted in strict accordance with international ethical standards and the principles of the Declaration of Helsinki. It adhered to the regulations set out in Mexico’s General Health Law on Health Research. The study protocol was reviewed and approved by the Institutional Research and Ethics Committees (approval ID ENFER-FACTOR-07 and CEICANCL-12052023). All participants provided written informed consent prior to taking part, and the anonymity and confidentiality of the data were strictly guaranteed. Data was collected by the research team through an online self-report survey. There was no economic reward included. However, upon survey completion, participants received an information sheet listing counseling services and resources for mental health support.

### 2.5. Data Analysis

All statistical analyses were conducted using SPSS version 29 (IBM Corp., Armonk, NY, USA). Descriptive statistics were applied to characterize the sample. For continuous variables, normality was tested, and depending on the distribution, either a Student’s *t*-test or a Mann–Whitney U test was used to compare groups. Data were reported as the mean ± standard deviation for continuous variables, and as frequencies (percentages) for categorical variables. For categorical variables, chi-square tests and odds ratios (OR) were calculated to assess associations with suicidal behavior. For categorical variables with three or more levels, post hoc procedures were conducted to identify the specific categories contributing to significant chi-square associations. Adjusted standardized residuals were examined for each cell, and Bonferroni-corrected pairwise chi-square tests were applied when appropriate. To identify independent risk factors, we performed multivariate logistic regression models, which provided adjusted odds ratios with 95% confidence intervals. Additionally, exploratory factor analysis (EFA) was conducted to explore latent dimensions among psychological and behavioral variables. Principal component analysis (PCA) was used for extraction, with factors selected according to eigenvalues greater than 1, and Varimax rotation was applied to enhance interpretability. Sampling adequacy was assessed using the Kaiser–Meyer–Olkin (KMO) measure and Bartlett’s test of sphericity. Statistical significance was set at *p* < 0.05 for all analyses.

To complement the primary inferential plan, we integrated the latent structure from the EFA with bivariate associations in a network graph. First, we computed point–biserial correlations between the dichotomous outcome (suicidal behavior: presence/absence) and each predictor. For interpretability and practical relevance, only links meeting two-sided *p* ≤ 0.05 and |r| ≥ 0.10 were retained. In parallel, we used the Varimax-rotated EFA results to assign each variable to the factor on which it showed the highest loading; factor links were drawn for loadings ≥ 0.40. Communalities were derived as the sum of squared loadings.

Networks were built in Python v. 3.11 (Wilmington, DE, USA) (*pandas*, *SciPy*, *NetworkX*, *Matplotlib*). Given the exploratory aim of the network, no multiple-testing adjustment was applied; all tests were two-sided (α = 0.05). For the comparative visualization, we additionally generated a companion network for medical students using the published Varimax loadings/communalities as priors, and reapplied the same correlation thresholds, weighting rules, and layout [55]. This side-by-side approach preserved the validated latent architecture while highlighting cohort-specific proximity to the outcome node, enabling a direct, interpretable comparison of convergences and divergences across disciplines.

## 3. Results

### 3.1. General Characteristics of the Study Population and Suicidal Behavior Stratification

The study sample consisted of 433 students enrolled in the Bachelor of Nursing program at the Autonomous University of Zacatecas. Most participants were female (77.8%) (Table 1). The mean age was 20.3 years (±2.78), with most participants aged between 18 and 23. In terms of gender identity, most students were categorized as cisgender (95.8%), with smaller proportions identifying as nonbinary (2.5%), transgender (1.2%), or pangender (0.5%). Regarding sexual orientation, 88.7% of students identified as heterosexual, 5.3% as bisexual, 2.8% as homosexual, and 3.2% as another minority orientation. Most students were single (93.5%), while very few were married (2.1%). Around 40.2% were from outside the local area, while 59.8% were residents. Fewer than one in three participants (28.2%) reported having paid employment during their studies, and nearly all respondents reported not having children (94.9%). Socioeconomic distribution showed that 59.8% were classified as middle class, 31.9% as lower middle class, 6.2% as upper middle class, and 2.1% as in poverty. Physical activity levels, as measured by the IPAQ, showed that 33.3% of participants reported high levels of activity, 37.9% medium levels, and 28.9% low levels.

Regarding suicidal behavior, the Beck Suicide Scale showed most students were classified as ‘normal’ (84.8%), while 10.6% required further assessment and 4.6% were classified as requiring treatment, suggesting clinical concern requiring immediate attention. Some participants reported a history of self-harm behaviors (15.2%), such as cutting (12.5%), burning (1.8%) or scratching with sharp objects (5.3%). The Beck Suicidal Ideation Scale cut-off point (present vs. absent) was used as a control to define the levels of suicidal behavior: subjects who scored in the range of ‘further evaluation or treatment recommended’ were categorized as with suicidal behavior, while those scoring in the ‘normal’ range were classified as individuals with absence of suicidal behavior. According to the above, when stratifying by suicidal behavior (ideation and/or attempts), significant associations were observed. Female sex (p = 0.014), foreigner status (*p* < 0.001), high impulsivity ($\chi^{2}\;=\;11.36$, p = 0.003) and severe hopelessness ($\chi^{2}\;=\;66.52$, p < 0.001) were associated with suicidal behavior. For foreigner status (students who relocated from another town or municipality within the country to attend the nursing program, rather than international students), the “Yes” category was less frequent among those with suicidal behavior (19.7% vs. 43.9%). Additionally, substance use, particularly alcohol and cannabis, was associated with a higher frequency of suicidal behavior (p < 0.05). In addition to the main sociodemographic factors, significant differences were found in three personal characteristics: sexual orientation, gender identity, and the presence of children. Students who identified as bisexual and those who identified as non-binary in terms of gender had a higher percentage of suicidal behaviors (*p* < 0.05). In a similar manner, students who had children were more likely to have suicidal thoughts or attempts than those who did not (*p* = 0.017).

To determine which categories contributed to the overall chi-squared patterns, the adjusted standardized residuals were examined and chi-squared tests with Bonferroni correction were applied. For the socioeconomic status categories (poverty, lower-middle, middle, and upper-middle), the overall chi-squared test was not significant ($\chi^{2}\;=\;2.20,\;p\;=\;0.33).$ None of the pairwise differences was significant following the Bonferroni correction, and no adjusted residual reached ±1.96. Regarding sexual orientation (heterosexual, bisexual, homosexual, and other minority orientations), the overall association approached significance $(\chi^{2}\;=\;2.15,\;p\;=\;0.14$). Adjusted residuals indicated a trend toward increased suicidal behavior among sexual minority students (with residual 2.26) and fewer cases among heterosexuals (with residual −2.26); however, these differences did not survive Bonferroni correction. The test showed no significant relationship between physical activity levels (low, moderate, or high; according to IPAQ classification) and suicidal behavior ($\chi^{2}\;=\;1.06,\;p\;=\;0.59$). Adjusted residuals were of modest magnitude (all less than or equal to ±1.0), confirming minimal differences across activity levels. Thus, no categorical predictors showed statistically significant post hoc differences after multiple-comparison correction. However, the pattern of residuals was consistent with descriptive trends indicating slightly elevated suicidal behavior among sexual minority students and those from lower socioeconomic backgrounds.

### 3.2. Mental Health Disorders

Considering ADHD, proportions of students classified as “ADHD indicative” were markedly higher in the suicidal-behavior group (63.6% vs. 39.8%) and “unlikely” correspondingly lower (16.7% vs. 33.0%; p <0.0001). Depression severity was also elevated, including a threefold excess of extremely severe cases (31.8% vs. 9.5%; p < 0.001). Anxiety displayed a similar gradient, with extremely severe symptoms roughly doubled (40.9% vs. 20.2%; p = 0.008).

Stress was higher among those with suicidal behavior (e.g., severe: 21.2% vs. 9.3%; no stress: 21.2% vs. 34.9%; p = 0.014). Hopelessness increased overall, with greater proportions reporting mild and moderate levels (33.3% and 10.6% vs. 14.4% and 0.5%; p = 0.001). By contrast (and contrary to expectation), high impulsivity was less frequent in the suicidal-behavior group (42.4% vs. 62.7%), while low impulsivity was more common (57.6% vs. 37.3%; p =0 .003). Anhedonia distributions did not differ significantly between groups (p = 0.123) (Table 2).

To elucidate relationships between psychological constructs and suicidal behavior, we conducted chi-square tests and post hoc analyses utilizing adjusted standardized residuals for multi-level severity variables. Among the constructs analyzed, only ADHD showed a statistically significant association with suicidal behavior ($\chi^{2}\;=\;18.77,\;p\;<\;0.001$). Post hoc inspection of adjusted residuals indicated that the *Probable ADHD* category was significantly overrepresented among students with suicidal behavior (Z = 3.30), while the same category was underrepresented among those without suicidal behavior (Z = −1.40), although this latter value did not exceed the standard threshold of ±1.96 for statistical significance. For depression, the overall chi-square test was also significant ($\chi^{2}\;=\;12.47,\;p\;=\;0.006$). Post hoc residuals showed that students with *no depression* were significantly underrepresented in the suicidal group (Z = −2.03), while no other severity levels showed significant deviations. In contrast, both the overall chi-square tests and post hoc residuals for anxiety and stress did not yield statistically significant differences between severity levels (p > 0.05 in both cases), suggesting no meaningful variation in suicidal behavior across those categories. Regarding hopelessness, the *Moderate* and *Severe* categories were significantly associated with greater suicidal behavior compared to the *No Hopelessness* group (p < 0.01), reinforcing its relevance as a risk factor. For impulsivity, a significant difference was observed between the *Low Impulsivity* and *High Impulsivity* groups. Students in the *Low Impulsivity* group were significantly more likely to report suicidal behavior (p = 0.003). Lastly, although anhedonia was measured across five frequency levels, the overall chi-square test did not show a statistically significant association with suicidal behavior (p = 0.123), and thus no post hoc analysis was warranted for this variable.

### 3.3. Substance Use and Use-Related Situations

Tobacco use differed significantly by suicidal status (χ^2^ test, *p* < 0.001): students exhibiting suicidal behavior displayed a higher proportion of mild–moderate tobacco use compared to their peers without such behavior (Table 3). Alcohol risk level was also associated with suicidal behavior (*p* = 0.001), with the medium- and high-risk categories being overrepresented among students with suicidal behavior, relative to those without. The ‘likely addiction’ category was rare overall, being observed only among participants without suicidal behavior (*n* = 8), which suggests sparse counts rather than a protective effect. Cannabis use was also significantly associated (*p* = 0.013), primarily due to a higher proportion of students with suicidal behavior endorsing symptoms of addiction compared with those without. The analysis of substance use situations, including pleasant emotions, testing self-control, physical discomfort, need/craving, unpleasant emotions, pleasant moments with others, conflict with others and social pressure, did not yield any statistically significant differences between groups (all *p* ≥ 0.120, with several comparisons showing *p* ≈ 0.43 to 1.00). These null findings likely reflect low base rates of situation-specific consumption in this cohort and limited statistical power to detect small between-group differences. Overall, the results suggest that substance use (tobacco, alcohol, cannabis) is more strongly linked to suicidal behavior in this sample than the specific contextual triggers in which consumption occurs.

### 3.4. Perceived Social Support

MOS inventory is a multidimensional instrument designed to assess the perceived availability of different types of social support. The four perceived social support dimensions derived from the MOS are material assistance (tangible support), emotional support, affective support, and social relations for leisure and distraction. Material assistance reflects the perceived availability of practical help; emotional support refers to access to empathy, understanding, and guidance; affective support captures expressions of affection and emotional closeness; and social relations indicate the availability of companionship for recreational or distracting activities.

The results of the MOS inventory are shown in Table 4. Significant differences were observed in the proportions of individuals with Maximum versus Minimum/Medium support across groups with and without suicidal behavior for all dimensions of the inventory (OR values ranging from 1.97 to 3.1; *p* < 0.001). For material assistance, 14 (4.8%) students perceived minimal support, 72 (24.5%) perceived medium support, and 208 (70.7%) perceived maximum support. In the emotional support section, 12 (4.1%) students were recorded as having minimal support, 74 (25.2%) as having medium support, and 208 (70.7%) as having maximum support. Affective support, relating to expressions of love and affection, 14 (4.8%) students perceived minimal support, 75 (25.5%) perceived medium support, and 205 (69.7%) perceived maximum support. For social relations of leisure and distraction, 12 (4.1%) students expressed a perception of minimal support, 71 (24.1%) expressed a perception of medium support, and 211 (71.8%) expressed a perception of maximum support. Significant differences were observed in the proportions of individuals with Maximum versus Minimum/Medium support across groups with and without suicidal behavior for all dimensions of the inventory (OR values ranging from 1.97 to 3.1; *p* < 0.001).

### 3.5. Multivariate Data Modeling to Weigh Variables Associated with Suicidal Behavior and Suicide Attempt

During the multivariate logistic regression analysis, the dichotomized Beck Suicide Scale was considered as the dependent variable, representing suicidal ideation and/or intent. Different sets of predictors were tested across five sequential models to determine the most stable and parsimonious ones (for additional information regarding data modeling, see Supplementary File S1, section Complementary Information: Multivariate data modeling).

Model 1 included thirteen sociodemographic, psychosocial and clinical variables (biological sex, sexual orientation, from another town, children, tobacco use, alcohol use, impulsivity, ADHD screening, hopelessness, emotional support, material help, social leisure/distraction, affective support). Hopelessness was strongly associated with suicidal behavior, with odds increasing by over tenfold (OR = 13.17; 95% CI: 2.34–74.27; *p* = 0.003). Emotional support was identified as a protective factor (OR = 0.019; 95% CI: 0.000–0.854, *p* = 0.041), whereas affective support exhibited an unexpectedly strong positive association (OR = 34.13; 95% CI: 1.05–1108.68, *p* = 0.047). However, the confidence intervals were extremely wide, reflecting overfitting and instability given the small sample size.

Model 2 comprised eleven clinical and demographic variables (age, biological sex, sexual orientation, socioeconomic status, anhedonia, impulsivity, ADHD screening, DASS-21 stress, DASS-21 depression, DASS-21 anxiety, severe self-harm [more than once]). Depression emerged as the primary significant factor, tripling the odds of suicidal behavior (OR = 2.99; 95% CI: 1.00–8.98; *p* = 0.050). Other variables, such as anxiety, stress, impulsivity and socioeconomic status, did not reach statistical significance in this configuration.

In Model 3, which focused on eight predictors combining social and clinical domains (sexual orientation, socioeconomic status, impulsivity, DASS-21 depression, hopelessness, severe self-harm, emotional support, affective support), both socioeconomic status and hopelessness reached statistical significance. Poorer socioeconomic status was associated with a markedly increased risk (OR = 20.50; 95% CI: 1.87–224.47; *p* = 0.013), while hopelessness again demonstrated a significant effect (OR = 5.95; 95% CI: 1.21–29.14; *p* = 0.028). Depression remained positive but lost significance in this set (*p* = 0.102).

In Model 4, which was reduced to five predictors (hopelessness, depression, socioeconomic status and emotional support), the model achieved greater stability. Both hopelessness (OR = 2.20; 95% CI: 1.30–3.71; *p* = 0.003) and depression (OR = 1.83; 95% CI: 1.15–2.91; *p* = 0.011) were statistically significant. The other predictors maintained their expected directions but did not reach significance.

The last one, Model 5, included only two predictors: hopelessness and depression. This parsimonious model was the most robust, with narrow confidence intervals and highly significant effects for both predictors (Table 5). Each unit increase in hopelessness doubled the odds of suicidal behavior (OR = 2.24; 95% CI: 1.43–3.50; *p* < 0.001), while each unit increase in depression increased the odds by 54% (OR = 1.54; 95% CI: 1.20–1.97; *p* < 0.001). Impulsivity showed a significant bivariate difference (Fisher’s exact test, *p* = 0.003) and was explored in earlier, broader blocks; however, it did not remain significant after adjustment and was therefore excluded from Model 4 to enhance parsimony. In sensitivity analyses that included impulsivity, effect directions for the core predictors were unchanged and fit indices showed negligible differences, supporting the decision to streamline.

To evaluate the performance of the five models, we calculated McFadden’s pseudo R^2^, along with AIC statistics (see Supplementary File S1, Table S2). Model 1 showed McFadden $R^{2}=\;0.188,\;\;AIC=\;100.41$), an acceptable fit but overfitting possibility. For model 2 ($R^{2}\;=\;0.183$) with an AIC = 85.56. Model 3 ($R^{2}=\;0.475;\;AIC\;=\;49.30$) achieved the best fit among the small-sample blocks. Model 4 reached pseudo $R^{2}=0.156$, with AIC= 153.83. Model 5 (which was most parsimonious in only including depression and hopelessness) carried a very reasonable $R^{2}$ of 0.155 and AIC (221.66), indicating that it had the best trade-off between model simplicity and explanatory power (Table S2. Multivariate models with adjusted odds ratio).

### 3.6. Exploratory Factor Analysis for Interdependence Between Variables

EFA was conducted exclusively among students who presented suicidal behavior, with the aim of identifying the latent structure underlying the psychological and contextual variables within this highest-risk subgroup. This approach made it possible to examine how key clinical and social indicators cluster and interact in students experiencing suicidal behavior. The resulting latent dimensions offer theoretical relevance for the present investigation and provide a foundation for future intervention design. Overall, the EFA revealed meaningful underlying constructs related to psychological distress and social protection in nursing students, which although presented briefly here, warrant further, more detailed analysis.

After examining the factor loading and communalities, three elements of theoretical coherence were identified. The first factor categorized internalizing symptoms, including depression, anxiety, stress, hopelessness, or ADHD indicators, indicating that there exists a latent construct best described as psychological vulnerability. This dimension mirrors the clustering of affective dysregulation and cognitive symptoms known to have predictive validity regarding suicidal risk in young adults. The second factor included impulsivity and substance-related behaviors, a concept that ties into behavioral dyscontrol. This grouping aligns with prior research that demonstrates that impulsivity and maladaptive coping (e.g., tobacco or cannabis use) increase suicide risk in emotionally vulnerable people. The third factor which strongly loaded was the perceived social support, which involved emotional, material, affective and leisure-oriented dimensions. This was considered a protective social buffering factor. Significantly, higher scores on this factor were associated with decreased suicidal ideation, consistent with established models of social connectedness as a buffer against emotional crises. Collectively, these three variables establish a conceptual pathway linking pathways of clinical risk and resilience.

The final model retained four factors extracted via principal components analysis with Varimax rotation, converging in five iterations. Sampling adequacy was satisfactory (KMO = 0.714) and Bartlett’s test of sphericity was significant ($\chi^{2}\;=\;350.29,\;df\;=\;91,\;p\;<\;0.001$), indicating that the correlation matrix was suitable for factor analysis (Table 6). Although limited from consideration in these articles, these findings could form the basis for a separate psychometric study examining how latent vulnerabilities and protections interact to shape suicidal trajectories in university populations.

The rotated solution revealed the following dimensions:

1. Situational Distress (SDC factor): This dimension grouped items related to emotional and interpersonal strain in consumption contexts, including unpleasant emotions (0.889), physical discomfort (0.915), the need or temptation to consume (0.870), conflict with others (0.937) and social pressure (0.872).
2. Clinical Distress (DASS/ADHD factor): This dimension captured core psychopathological symptoms, including stress (0.786), depression (0.917), anxiety (0.930), ADHD screening (0.788) and anhedonia (0.609).
3. Cognitive–emotional vulnerability: This factor was primarily defined by hopelessness (0.787) and anhedonia (0.564), with moderate contributions from living context (0.486) and hailing from a different town (−0.750). It reflects a latent vulnerability pattern combining negative cognitive and emotional states with contextual fragility.
4. Social identity/Context: This dimension grouped social background variables, including sexual orientation (0.849) and living arrangements (0.752).

### 3.7. Network Analysis and Factorial Visualization

As a complementary procedure to the primary statistical analyses, a computational approach was developed to represent the multivariate structure of psychosocial and clinical predictors of suicidal behavior through a network graph. The process integrated correlation analysis, factorial loadings, and graph theory to provide a comprehensive and visual framework for interpreting the interrelations among variables. Point-biserial correlations were first calculated between the dichotomous dependent variable (Beck Suicide Scale, coded as presence or absence of suicidal risk) and each of the independent variables. To ensure both statistical and practical relevance, only associations that met the thresholds of *p* ≤ 0.05 and |r| ≥ 0.10 were retained. In parallel, factorial loadings obtained from a previous Varimax-rotated exploratory factor analysis were incorporated.

**Figure 1:**
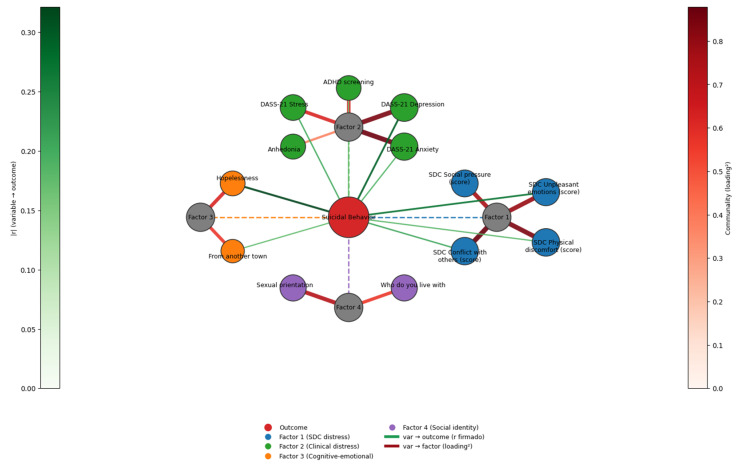
Graphical integration of correlations and factorial loadings in relation to suicidal behavior in nursing students. The network illustrates the structure of the psychosocial and clinical predictors of suicidal behavior among nursing students. The central red node represents the dichotomous outcome, ‘Suicidal Behavior’ and is connected with dotted lines to the identified factors (gray nodes). The gray nodes represent the four latent factors obtained via Varimax-rotated exploratory factor analysis, while the colored peripheral nodes represent the observed variables. Edges from the observed variables to the outcome node represent statistically significant point-biserial correlations (*p* ≤ 0.05 and r ≥ 0.10). Their thickness is proportional to r and the green color scale on the left-hand side of the figure encodes the same magnitude. Edges from variables to factors are drawn when the loading is ≥0.40 and reflect squared loadings; their thickness is proportional to their contribution to communality, and the red color scale on the right-hand color bar encodes that value. Node sizes are scaled by each variable’s total communality and node colors indicate factor membership. Dashed outcome-to-factor edges are visual guides only. In a radial layout, the outcome is placed at the center, the factors on an intermediate ring and the variables on outer arcs. Variables lacking both sufficient loading and a significant correlation with the outcome are omitted for clarity.

Four latent dimensions had been identified in this analysis, corresponding to situational distress, clinical distress, cognitive–emotional vulnerability, and social identity/context. Each variable was assigned to the factor on which it exhibited its strongest loading, and measures of communality and squared loading contributions were derived to quantify the extent of variance explained. The implementation was carried out in Python (see Section 2 for more information). The graph was composed of three categories of nodes: the central outcome (suicidal behavior), the four latent factors, and the observed variables (Figure 1).

Correlation-based edges were drawn between variables and the outcome, with their width scaled to the absolute correlation and their color mapped according to the sign of the association. Factorial edges linked each variable to its corresponding factor, weighted by the squared loading as a representation of communality. The network was arranged in a radial layout, positioning the outcome node at the center, the four latent factors evenly distributed in a surrounding layer, and the variables located on a peripheral ring according to their assigned factor. Node sizes were also scaled proportionally to communalities, and colors were used to distinguish between factors, thus enhancing interpretability of the visualization. The graph was exported as a high-resolution image file, while a complementary table was produced containing correlation coefficients, factor assignments, and communalities for each variable. This methodological addition integrates conventional statistical outputs with a graphical exploration, enabling a clearer understanding of the latent structure and relational patterns that underlie suicidal behavior among nursing students (Figure 1).

## 4. Discussion

In this study, by using validated inventories and a combined statistical approach involving bivariate analyses, multivariate logistic regression and exploratory factor analysis, the psychosocial context and risk and protective factors contributing to suicidal behavior in undergraduate nursing students were evaluated. In the bivariate analyses, several demographic, psychosocial, and behavioral differences were observed between students with and without suicidal behavior. Higher prevalence was found among women, students with non-heterosexual orientations or nonbinary identities, and among those who were married/in a relationship or who had children, consistent with evidence on minority stress and the additional demands associated with caregiving roles [27,56]. Regarding geographical origin, the “foreigner” variable referred to students from municipalities outside the city of Zacatecas. Local students showed higher suicidal behavior, possibly reflecting stronger family pressures or lower perceived autonomy. Psychologically, students with suicidal behavior had higher levels of ADHD symptoms, depression, anxiety, stress, impulsivity, and hopelessness [17,18]. For behavioral factors, cannabis and tobacco use were significantly associated with suicidal behavior, even though situational use was not. Prior studies indicate that regular use of these substances may increase depressive and anxious symptoms and reflect dysfunctional coping with academic or emotional stress [57]. Thus, substance use may act as a general indicator of psychological distress, supporting the need for prevention and consumption-reduction strategies in nursing education [58].

While initial models indicated that multiple factors were relevant in bivariate tests, only two psychological constructs, hopelessness and depression, remained stable predictors across successive multivariate models. The final parsimonious model revealed a strong association between hopelessness and suicidal behavior, with the odds of the outcome being more than doubled (OR = 2.24, 95% CI: 1.43–3.50, *p* < 0.001). Meanwhile, depressive symptoms were found to increase the odds by 54% (OR = 1.54, 95% CI: 1.20–1.97, *p* < 0.001). These findings are consistent with longitudinal evidence showing that hopelessness and depression are reliable predictors of suicidal thoughts, attempts and death [59,60]. Although variables such as tobacco and cannabis use, among others, showed significant associations with suicidal behavior in the bivariate analyses, these variables did not retain significance in the multivariate models. This pattern is consistent with prior literature indicating that the relationship between substance use and suicidal ideation in young adults often becomes nonsignificant after adjusting for depressive symptoms or other psychological mediators.

To complement the regression models and assess the broader latent dimensions in which these predictors are embedded, an exploratory factor analysis was conducted. The rotated solution yielded a four-factor structure that accounted for the main areas associated with suicidal behavior:

1. Situational Distress (SDC factor): This dimension reflected the role of consumption and interpersonal strain, with high loadings on unpleasant emotions (0.889), physical discomfort (0.915), temptation to consume (0.870), conflict with others (0.937) and social pressure (0.872). These findings support the idea that situational triggers, particularly those linked to social stress and substance use, can lead to suicidal behavior [57].
2. Clinical Distress (DASS/ADHD factor): This dimension grouped core psychopathological indicators, including stress (0.786), depression (0.917), anxiety (0.930), ADHD-related traits (0.788) and anhedonia (0.609). Clustering these variables shows the role of comorbid psychiatric conditions in elevating suicide risk. This is consistent with extensive literature linking mood and attention disorders to suicidal behavior [58,61].
3. Cognitive–emotional vulnerability: This factor was defined by hopelessness (0.787) and anhedonia (0.564), with a contextual contribution from living arrangements (0.486). The co-occurrence of these elements reflects a vulnerability structure characterized by pessimistic cognitions, diminished capacity for positive effects and contextual fragility. These are all recognized as proximal risk markers for suicide [62].
4. Social identity/context: This dimension encompasses sexual orientation (0.849), living arrangements (0.752) and geographic origin (–0.750 for those from another town). This is consistent with evidence that sexual minorities and socially isolated students are disproportionately vulnerable to mental health issues and suicidal behavior due to the combined effects of stigma and discrimination, as well as having limited access to protective social networks [56].

Finding the psychological and contextual predictors of suicidal conduct in nursing students was one of the study’s goals, and the results are in agreement with those goals. According to the original predictions, perceived social support would serve as a protective factor, while depressive and hopeless symptoms would be the primary predictors of suicide risk. The findings mainly corroborate these theories, demonstrating that suicidal behavior is buffered by affective and emotional support, while despair and hopelessness have the highest predictive weight. According to Beck’s cognitive-behavioral and hopelessness models, these findings support the central role of negative emotional factors as axes of suicide risk [63].

**Figure 2:**
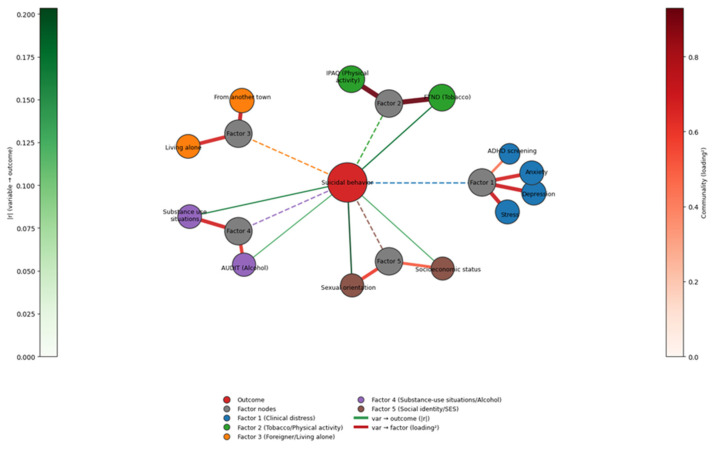
Graphical integration of correlations and factorial loadings in relation to suicidal behavior in medical students. The network illustrates the multivariate structure of the psychosocial and clinical predictors of the dichotomous outcome, suicidal behavior (the central red node) and is connected with dotted lines to the identified factors. Gray nodes represent the five latent factors derived from the Varimax-rotated component matrix: Factor 1: Clinical distress; Factor 2: Tobacco/physical activity; Factor 3: Foreigner/living alone; Factor 4: Substance use/alcohol; Factor 5: Social identity/SES. The colored peripheral nodes represent observed variables that have been assigned to factors based on their highest loading. Green edges leading to the outcome node indicate statistically significant point-biserial correlations (*p* ≤ 0.05 and |r| ≥ 0.10). The thickness of these edges and the scale of the left green color bar are proportional to the magnitude of the correlation coefficient (r). Red edges to factor nodes encode squared loadings (loading^2^), with thickness proportional to communality, and the right-hand red color bar indicating its scale. Node sizes reflect total communality and node colors distinguish factor membership. The radial layout places the outcome at the center, the factors on an intermediate ring and the variables on the periphery. Dashed links from the outcome to the factor nodes are visual guides only. Variables that do not meet the correlation or loading thresholds are omitted for clarity.

With the aim of performing a comparison with previous studies in similar populations, we drew on the empirical structure reported by Martinez-Fierro et al. (2025) in a cross-sectional cohort study of Mexican medical students [55]. This study identified the latent components and multivariate associations underlying suicidal behavior. Using these published results of factor analysis as priors, we decided to construct a corresponding network graph for the medicine cohort and place it alongside the nursing cohort graph. This approach enabled us to preserve the validated latent architecture (via rotated loadings/communalities) while visualizing cohort-specific proximities to the suicidal behavior node (via point-biserial and logistic links). This allowed us to make a direct, interpretable comparison of convergences and divergences across disciplines. In both cohorts, the overall architecture is consistent with a hub-and-spoke representation (Figure 2). When comparing results in nursing students with those obtained in medical students, important similarities but also marked differences in the architecture of suicide risk in both groups can be observed. In both cohorts, a common core of clinical distress (depressive-anxious symptoms, stress, impulsivity, etc.) strongly linked to suicidal behavior was identified, confirming that the burden of internal psychopathology (e.g., depression, anxiety) is a key determinant of risk in health science students in general. However, contextual and identity factors were organized differently between the two programs. Among medical students, variables such as tobacco use, physical activity, being a foreigner (coming from another city), living alone, and minority sexual orientation tended to cluster into several distinct components, each with high internal consistency but relatively weak direct associations with suicidal behavior (bivariate correlations around r ≈ 0.20). In contrast, among nursing students, the structure was more compact: the situational variables of substance use and interpersonal conflict were concentrated in a single contextual distress factor (the SDC factor described above) and showed stronger direct links to suicidal behavior (correlations of up to r ≈ 0.30). In the nursing cohort, therefore, acute situational stress associated with substance use and interpersonal problems is more closely related to suicidal ideation or attempts, while in medicine, these same factors operate more indirectly or distally. These contrasts probably reflect differences in the training demands and experiences of each discipline. The literature reports that, due to an intense curriculum with clinical rotations and early care responsibilities, nursing students often experience very high levels of stress during their training [64,65].

This could explain why, in our study, immediate situational stressors (linked to clinical practices or the social environment) had greater weight in this group. Medical students, on the other hand, often face high levels of academic pressure and, in many cases, geographical relocation and social isolation when entering the program; such long-term contextual factors could influence their mental health in a more diffuse or time-mediated way. Consistent with this, different points of preventive intervention emerge for each population: our data suggest that, among nursing students, it would be a priority to address acute situational distress (e.g., through training in stress management during clinical practice, attention to interpersonal conflicts, and substance abuse prevention), while among medical students, greater emphasis may be needed on strategies that address chronic contextual factors (e.g., support programs for foreign or isolated students, promotion of social support networks, and management of prolonged academic stress).

In line with these structural differences, multivariate analyses of both cohorts show contrasting patterns. In medical students, recent evidence indicates that, in addition to hopelessness, several contextual conditions have an independent effect on suicidal behavior. For example, Martinez-Fierro et al. (2025) reported that factors such as tobacco use, alcohol consumption, family history of mental illness, and even lack of material/emotional support were significantly associated with increased suicide risk in multivariate analysis (odds ratios between ~1.5 and 8.8, *p* < 0.05) [55]. In contrast, in the nursing cohort of the present study, none of these contextual factors retained statistical significance when hopelessness and depression were simultaneously included in the model. In other words, in nursing students, the negative emotional core (hopelessness/depression) absorbed virtually all the explanatory power of the model, relegating contextual factors to an indirect role. This suggests that in this population, contextual factors could influence suicide risk by mediating through psychological state (for example, an environment with little social support could lead to hopelessness, which is what ultimately triggers suicidal behavior). In contrast, among medical students, these external factors would retain a more direct role in risk, perhaps because the heterogeneity of their sample and greater statistical power allowed their independent effects to be detected. These observations highlight the importance of considering the disciplinary context when analyzing suicidal behavior: even within populations of similar age and educational level, the configuration of risk factors may differ, and therefore interventions must be adapted to these particularities.

In terms of protective factors, the role of perceived social support and its differences between cohorts deserves attention. Among medical students, greater material and emotional support from family or friends was associated with a lower likelihood of suicidal behavior, even after adjusting for other factors in the multivariate model [55]. This supports the abundant evidence on the protective role of strong social support networks for the mental health of university students. However, in the nursing cohort, although lower rates of suicide risk were observed among those who reported strong social support in the bivariate analyses (e.g., those with high material and emotional support had a lower prevalence of ideation/attempts), social support did not retain a significant association in the final multivariate models. This finding suggests that the beneficial effect of social support in nursing manifests indirectly, possibly by buffering the development of hopelessness or depression, rather than as an isolated factor with a direct effect on suicidal behavior. This is consistent with what was reported in the medical cohort, where certain dimensions of support (such as emotional support) also showed no direct effect after controlling for other risks [55].

In a general manner, our results are in line with those of worldwide studies on nursing students, which found that the most reliable indicators of suicidal thoughts and actions are depression and hopelessness [63]. Comparable patterns of psychological vulnerability have also been linked in earlier research to inadequate social support, academic overload, and insufficient coping mechanisms [66]. Conversely, some authors have emphasized the significance of contextual factors that have more immediate impacts than those discovered in our research [67]. These differences could be due to the cross-sectional nature of the present study and cultural variability between educational contexts. From an applied perspective, the results highlight the need to implement institutional suicide prevention strategies in nursing schools, focusing on the early detection of symptoms of hopelessness and depression. In line with international efforts, suicide prevention interventions have been developed even for nurses graduating from university, such as the Healer Education Assessment and Referral (HEAR) program, which provides education on suicide risk factors, anonymous risk detection, and referral for further treatment [68]. Moreover, interventions tailored to students have also been implemented globally, commonly incorporating training programs for guardians or counselors, as well as initiatives aimed directly at enhancing students’ mental health literacy and coping abilities [69]. The integration of mental health promotion programs, emotional coping workshops, and peer support networks could contribute to reducing the incidence of suicidal ideation.

### Study Limitations and Future Directions

It is important to recognize the limitations of this study when interpreting the results. The cross-sectional design prevents us from establishing causal relationships; we cannot be sure whether factors such as hopelessness lead to suicidal behavior or whether, conversely, suicidal ideation intensifies feelings of hopelessness (most likely, there is a bidirectional, feedback relationship). Future longitudinal studies would be valuable in elucidating the temporal sequence between the identified variables and the development of suicidal behaviors [55]. Second, the sample is limited to nursing students at a specific university, which limits the generalizability of the findings to other institutions or countries; local sociocultural factors could influence the prevalence and interaction of risks, so replicating this analysis in different academic contexts would be relevant. Another limitation is the use of self-report instruments, which may lead to social desirability bias or underreporting of sensitive behaviors; although validated and anonymized scales were used, there is always the possibility of information bias in this type of study. Nevertheless, this study also has several strengths: it included a large sample size, used internationally validated standardized instruments for a comprehensive assessment (from psychopathological symptoms to social support and lifestyle habits), and applied a multidimensional analytical approach that combined traditional models with network/factor analysis techniques. This integrated approach allowed us not only to identify individual predictors but also to visualize how they are interconnected within the student’s psychosocial framework, providing a richer picture for the interpretation of suicidal behavior in the university population. psychosocial framework, providing a richer picture for the interpretation of suicidal behavior in the university population.

Although academic stress was included as one of the measured psychological variables in this study, other stressors inherent to nursing education, such as clinical workload, exposure to patient suffering, and emotionally demanding practice scenarios, were not directly assessed. Future research should incorporate validated measures of these clinical training–related stressors, as they may interact with academic demands and individual psychological factors to further shape suicidal ideation and behavior in nursing students.

It is important to note, this analysis is the result of previous research carried out by the same authors, which focused on predictors of suicidal behavior in medical students (Martinez-Fierro et al., 2025) [55]. The analysis revealed three coherent latent constructs: (1) a dimension of psychological vulnerability defined by internalizing symptoms such as depression, anxiety, stress, hopelessness, and ADHD traits; (2) a behavioral dyscontrol factor grouping impulsivity and maladaptive substance use; and (3) a protective factor characterized by perceived social support across emotional, material, affective, and leisure dimensions. These factors were consistent with theoretical models of suicide risk and resilience and aligned with previous empirical frameworks in similar populations. While briefly presented in this study, these findings could serve as the conceptual foundation for a future psychometric investigation into how latent vulnerabilities and strengths shape suicidal trajectories in university contexts. Unlike that study, this analysis focuses on nursing students with the aim of establishing comparisons between patterns of protection and risk in different health disciplines. Although a similar methodological design was maintained to facilitate comparison, the statistical analysis, the population studied, and the interpretation from a theoretical perspective are unique to this research.

## 5. Conclusions

This study identified a multidimensional structure of suicidal behavior in nursing students and confirms that internal psychopathological burden (depression, anxiety, etc.) and hopelessness play a central role; on the other hand, it emphasizes that situational stressors (such as acute experiences of conflict or substance use) and conditions of social isolation or belonging to sexual minorities also form part of the risk framework, although their influence may vary between different degree programs. The latent dimensions—clinical distress, situational discomfort, cognitive–emotional vulnerability, and context/social identity—provide insight into how personal, contextual, and social factors interact in the development of suicidal ideation and attempts. Our findings support the need for comprehensive prevention strategies that address both emotional distress and acute stressors, as well as conditions of social isolation. While some risk factors are shared across health-related disciplines such as human medicine, the specific context of nursing education calls for tailored interventions. Routine population-based mental health screenings are recommended as part of systematic efforts to promote student well-being and prevent suicide risk in university settings.

## Figures and Tables

**Table 1:** General characteristics of the study population classified as presence and absence of suicidal behavior.

Variable	Total (*n* = 433)	Suicidal Behavior		*p*-Value
		Presence (*n* = 66)	Absence (*n* = 367)	
**Age**	20.3 ± 2.78	20.7 ± 3.03	20.2 ± 2.74	0.237
**Sex**				**0.015**
Female	337 (77.8)	59 (89.4)	278 (75.7)	
Male	96 (22.2)	7 (10.6)	89 (24.3)	
**Foreigner**				**<0.001**
No	259 (59.8)	53 (80.3)	206 (56.1)	
Yes	174 (40.2)	13 (19.7)	161 (43.9)	
**Social class**				0.095
Poverty	9 (2.1)	4 (6.1)	5 (1.4)	
Lower middle class	138 (31.9)	21 (31.8)	117 (31.9)	
Middle class	259 (59.8)	38 (57.6)	221 (60.2)	
Upper middle class	27 (6.2)	3 (4.5)	24 (6.5)	
**Living alone**				-
No	433 (100)	66 (100)	367 (100)	
Yes	0 (0)	0 (0)	0 (0)	
**Sexual orientation**				**0.028**
Heterosexual	384 (88.7)	56 (84.8)	328 (89.4)	
Bisexual	23 (5.3)	8 (12.1)	15 (4.1)	
Homosexual	12 (2.8)	0 (0.0)	12 (3.3)	
Other	14 (3.2)	2 (3)	11 (4.7)	
**Gender identity**				**0.027**
Cisgender	415 (95.8)	61 (92.4)	354 (96.5)	
Nonbinary	11 (2.5)	5 (7.6)	6 (1.6)	
Pangender	2 (0.5)	0 (0.0)	2 (0.5)	
Transgender	5 (1.2)	0 (0.0)	5 (1.4)	
**Marital status**				**0.04**
Married	9 (2.1)	2 (3.0)	7 (1.9)	
Divorced	1 (0.2)	0 (0.0)	1 (0.3)	
In a relationship	4 (0.9)	2 (3.0)	2 (0.5)	
Single	405 (93.5)	59 (89.4)	346 (94.3)	
Cohabiting/Common-law union	13 (3.0)	2 (3.0)	11 (3.0)	
Widowed	1 (0.2)	1 (1.5)	0 (0.0)	
**Working**				1.0
No	311 (71.8)	48 (72.7)	263 (71.7)	
Yes	122 (28.2)	18 (27.3)	104 (28.3)	
**Children**				**0.017**
No	279 (94.9)	40 (87.0)	239 (96.4)	
Yes	15 (5.1)	6 (13.0)	9 (3.6)	
**Physical activity**				0.524
High	144 (33.3)	19 (28.8)	125 (34.1)	
Medium	164 (37.9)	29 (43.9)	135 (36.8)	
Low	125 (28.9)	18 (27.3)	107 (29.2)	

Age is reported as mean ± SD and compared with Welch’s *t*-test. Categorical variables are shown as *n* (%) and tested two-sided at α=0.05, Fisher’s exact test for 2 × 2 comparisons (biological sex, from another town, working status, children) and chi-square tests for variables with more than two categories (socioeconomic status, gender identity, marital status, IPAQ physical activity). Sexual orientation was analyzed in four categories (heterosexual, bisexual, homosexual, and “Other,” with “Other” collapsing asexual, autosexual, demisexual, and pansexual to ensure stable expected count) and was evaluated with a chi-square test. “Living alone” was not tested due to no variability (all responses “No”). Note: p-values reflect chi-square tests comparing the proportion of students with suicidal behavior between the groups indicated.

**Table 2:** Results of the mental health inventories administered to the nursing students classified according to presence or absence of suicidal behavior.

Variable	Total (*n* = 433)	Suicidal Behavior		*p*-Value
		Presence (*n* = 66)	Absence (*n* = 367)	
**ADHD**				
Unlikely	132 (30.5)	11 (16.7)	121 (33.0)	**<0.0001**
Probable	113 (26.1)	13 (19.7)	100 (27.2)	
Indicative	188 (43.4)	42 (63.6)	146 (39.8)	
**Depression**				
No depression	106 (24.5)	8 (12.1)	98 (26.7)	**<0.001**
Mild	26 (6.0)	1 (1.5)	25 (6.8)	
Moderate	64 (14.8)	6 (9.1)	58 (15.8)	
Severe	42 (9.7)	10 (15.2)	32 (8.7)	
Extremely severe	56 (12.9)	21 (31.8)	35 (9.5)	
**Anxiety**				
No anxiety	91 (21.0)	9 (13.6)	82 (22.3)	**0.008**
Mild	17 (3.9)	1 (1.5)	16 (4.4)	
Moderate	49 (11.3)	4 (6.1)	45 (12.3)	
Severe	36 (8.3)	5 (7.6)	31 (8.4)	
Extremely severe	101 (23.3)	27 (40.9)	74 (20.2)	
**Stress**				
No stress	142 (32.8)	14 (21.2)	128 (34.9)	**0.014**
Mild	33 (7.6)	4 (6.1)	29 (7.9)	
Moderate	47 (10.9)	7 (10.6)	40 (10.9)	
Severe	48 (11.1)	14 (21.2)	34 (9.3)	
**Impulsivity**				
High impulsivity	258 (59.6)	28 (42.4)	230 (62.7)	**0.003**
Low impulsivity	175 (40.4)	38 (57.6)	137 (37.3)	
**Hopelessness**				
No hopelessness	174 (40.2)	28 (42.4)	146 (39.8)	**0.001**
Mild	75 (17.3)	22 (33.3)	53 (14.4)	
Moderate	9 (2.1)	7 (10.6)	2 (0.5)	
Severe	174 (40.2)	28 (42.4)	146 (39.8)	
**Anhedonia**				
Always	67 (15.5)	10 (15.2)	57 (15.5)	0.123
Almost always	197 (45.5)	22 (33.3)	175 (47.7)	
Sometimes	83 (19.2)	19 (28.8)	64 (17.4)	
Rarely	42 (9.7)	6 (9.1)	36 (9.8)	
Never	67 (15.5)	10 (15.2)	57 (15.5)	

Differences were tested two-sided at α = 0.05 using chi-square tests of independence for multi-level variables (ADHD, Depression, Anxiety, Stress, Hopelessness, Anhedonia) and Fisher’s exact test for the 2 × 2 table (Impulsivity). p-values < 0.05 are highlighted in bold.

**Table 3:** Comparisons of substance consumption patterns and situations between individuals with presence and absence of suicidal behavior.

Variable	Total (*n* = 433) *	Suicidal Behavior		*p*-Value
		Presence (*n* = 66)	Absence (*n* = 367)	
**Tobacco**				
Without consumption	75 (17.3)	23 (34.8)	52 (14.2)	**<0.001**
Mild	23 (5.3)	7 (10.6)	16 (4.4)	
Moderate	4 (0.9)	1 (1.5)	3 (0.8)	
**Alcohol**				
Low risk	180 (41.6)	26 (39.4)	154 (42.0)	**0.001**
Medium risk	82 (18.9)	22 (33.3)	60 (16.3)	
High risk	11 (2.5)	4 (6.1)	7 (1.9)	
Likely addiction	8 (1.8)	0 (0.0)	8 (2.2)	
**Cannabis**				
Without symptoms of addiction	24 (5.5)	8 (12.1)	16 (4.4)	**0.013**
With symptoms of problematic use	13 (3.0)	2 (3.0)	11 (3.0)	
Symptoms of addiction	11 (2.5)	4 (6.1)	7 (1.9)	
*Substance use situations*				
1. **Pleasant emotions**				
Indicates consumption	12 (9.8)	1 (3.8)	11 (11.5)	0.432
Did not use the substance	110 (90.2)	25 (96.2)	85 (88.5)	
2. **Self-control**				
Indicates consumption	6 (4.9)	2 (7.7)	4 (4.2)	0.821
Did not use the substance	116 (95.1)	24 (92.3)	92 (95.8)	
3. **Physical discomfort**				
Indicates consumption	10 (8.2)	1 (3.8)	9 (9.4)	0.611
Did not use the substance	112 (91.8)	25 (96.2)	87 (90.6)	
4. **Need or craving**				
Indicates consumption	3 (2.5)	0 (0.0)	3 (3.1)	0.842
Did not use the substance	119 (97.5)	26 (100.0)	93 (96.9)	
5. **Unpleasant emotions**				
Indicates consumption	12 (9.8)	1 (3.8)	11 (11.5)	0.611
Did not use the substance	110 (90.2)	25 (96.2)	85 (88.5)	
6. **Pleasant moments with others**				
Indicates consumption	19 (15.6)	1 (3.8)	18 (18.8)	0.120
Did not use the substance	103 (84.4)	25 (96.2)	78 (81.2)	
7. **Conflict with others**				
Indicates consumption	8 (6.6)	1 (3.8)	7 (7.3)	0.855
Did not use the substance	114 (93.4)	25 (96.2)	89 (92.7)	
8. **Social pressure**				
Indicates consumption	8 (6.6)	2 (7.7)	6 (6.2)	1.00
Did not use the substance	114 (3.4)	24 (92.3)	90 (93.8)	

* Percentages are based only on participants who reported use of the respective substance; non-users were not included in the denominator (FTND [tobacco]: 331, AUDIT [alcohol]: 152, cannabis: 385, cocaine: 432). The percentages in the “Total (n = 433) (%)” column are calculated against the full sample (n = 433) rather than only respondents to each instrument. Consequently, category percentages appear small and do not add to 100% within an instrument, and they should be interpreted as prevalence in the entire cohort, not as within-instrument distributions. Note: In addition to the overall chi-square comparisons, we conducted post hoc analyses using adjusted standardized residuals to explore which specific categories contributed to significant associations. For alcohol use, the overall chi-square was significant ($\chi^{2}\;=\;9.88,\;p\;=\;0.020$), and post hoc residuals revealed a trend toward overrepresentation of suicidal behavior among students in the medium risk category (Z = 1.75), though this did not reach the conventional threshold for statistical significance (|Z| ≥ 1.96). For tobacco and cannabis use, neither the overall chi-square nor the post hoc residuals revealed meaningful differences between consumption levels. These findings suggest that while alcohol risk level may relate to suicidal behavior, particularly at moderate levels, other substance-related categories did not demonstrate significant subgroup variation. Percentages in the “Total (*n* = 433)” column are calculated against the full sample, and not within instrument-specific users. p-values < 0.05 are highlighted in bold.

**Table 4:** Social support findings and their comparison between nursing students with and without suicidal behavior.

Variable	Total (*n* = 433)	Suicidal Behavior		OR(95%CI)	*p*-Value
		Presence (*n* = 66)	Absence (*n* = 367)		
**Material assistance**					**<0.0001**
Maximum	208 (70.7)	15 (46.9)	184 (74.2)	2.68 (1.84–3.9)	
Medium	72 (24.5)	14 (43.8)	54 (21.8)		
Minimum	14 (4.8)	3 (9.4)	10 (4.0)		
**Emotional support**					**<0.0001**
Maximum	208 (70.7)	15 (32.6)	193 (72.3)	2.1 (1.45–2.92)	
Medium	74 (25.2)	14 (41.3)	60 (22.5)		
Minimum	12 (4.1)	5 (26.1)	7 (2.6)		
**Affective support**					
Maximum	205 (69.7)	19 (50.0)	186 (73.5)	3.1 (2.03–4.59)	**<0.0001**
Medium	75 (25.5)	12 (31.6)	63 (24.9)		
Minimum	14 (4.8)	7 (18.4)	7 (2.8)		
**Social relations of leisure and distraction**					
Maximum	211 (71.8)	17 (36.2)	194 (77.7)	1.97 (1.31–2.94)	**0.0013**
Medium	71 (24.1)	21 (44.7)	50 (20.0)		
Minimum	12 (4.1)	9 (19.1)	3 (1.2)		

Values are n (%) for maximum, medium, and minimum support across four subscales (material assistance, emotional support, affective support, and social relations-leisure/distraction) and for groups with vs. without suicidal behavior (Presence n=66; Absence n=367). Odds ratios (OR) compare Maximum vs. Medium/Minimum support. Percentages are calculated on available responses (valid n varies by subscale), so totals may not correspond to 433. Group differences were tested with chi-square; OR (95% CI) and p<0.05 are highlighted in bold. Note: In addition to the overall chi-square comparisons, we performed post hoc analyses using adjusted standardized residuals to determine which specific support categories contributed to the observed associations. For emotional support, both medium (Z = 1.86) and minimum support (Z = 3.35) were over-represented among students with suicidal behavior, with minimum support exceeding the significance threshold (Z > 1.96). Similarly, for affective support, the minimum support group showed a strong overrepresentation in the suicidal group (Z = 3.13). In contrast, students in the maximum support category were underrepresented among those with suicidal behavior. For material assistance, medium support was marginally over-represented (Z = 2.23), though not all comparisons reached significthough not all comparisons reached significance. For social leisure relations, minimum support again showed significant overrepresentation in the suicidal group (Z = 3.89). These results indicate that lower levels of perceived support across all four subdomains are associated with increased suicidal behavior risk. Percentages are calculated based on valid responses per subscale. p-values < 0.05 are bolded. Odds ratios compare maximum vs. medium/minimum levels.

**Table 5:** Multivariate logistic regression analysis of variables associated with suicidal behavior.

Predictor	*p*-Value	OR	95% CI
Hopelessness (score)	**<0.001**	2.24	1.43–3.50
DASS-21 Depression (score)	**<0.001**	1.54	1.20–1.97

OR = Odds Ratio; CI = Confidence Interval; p = significance level. Dependent variable: Beck Suicide Scale (dichotomized). *p* < 0.5 are highlighted in bold.

**Table 6:** Communalities obtained using principal component analysis.

Variable	Component			
	1	2	3	4
SDC Conflict with others	**0.937**	0.119	0.081	−0.098
SDC Physical discomfort	**0.915**	0.277	−0.014	−0.084
SDC Unpleasant emotions	**0.889**	0.278	0.032	−0.125
SDC Social pressure	**0.872**	0.181	−0.058	0.263
SDC Need or temptation	**0.870**	0.262	0.062	0.186
DASS-21 Anxiety	0.171	**0.93**	0.042	0.021
DASS-21 Depression	0.299	**0.917**	0.022	−0.021
ADHD screening	0.260	**0.788**	0.043	0.068
DASS-21 Stress	0.319	**0.786**	−0.028	0.229
Anhedonia	0.039	**0.609**	**0.564**	−0.153
Hopelessness	0.098	0.268	**0.787**	−0.058
Who do you live with	0.042	0.021	**0.486**	**0.752**
Sexual orientation	0.005	0.106	−0.281	**0.849**
From another town	0.044	0.18	**−0.750**	−0.051

Only cases where the suicidal behavior value equals 1 were used in the analysis phase. Extraction method: Principal components analysis. Rotation method: Varimax with Kaiser normalization. Rotation converged in 5 iterations. Only cases with the Beck Suicide Scale (interpretation) dichotomized = 1 were included. Values in bold indicate absolute rotated component loadings greater than 0.5. SDC: Substance use situations.

## Data Availability

The original contributions presented in this study are included in the article/Supplementary Materials. Further inquiries can be directed to the corresponding authors.

## Supplementary Materials

The following supporting information can be downloaded at: https://www.mdpi.com/article/10.3390/nursrep15120441/s1, Supplementary File S1: Table S1. Reliability and validity of psychological and substance use instruments used in the study; Complementary information: Multivariate data modeling. Table S2. Multivariate models with adjusted odds ratio.

## Public Involvement Statement

No public involvement in any aspect of this research.

## Guidelines and Standards Statement

This manuscript was drafted against The Strengthening the Reporting of Observational Studies in Epidemiology (STROBE) for observational research (https://www.equator-network.org/) (accessed on 1 July 2025).

## Abbreviations

The following abbreviations are used in this manuscript:

WHO	World Health Organization
OECD	Organization for Economic Cooperation and Development
ADHD	Attention-deficit/hyperactivity disorder
DASS-21	Depression, Anxiety and Stress Scale
IPAQ	International Physical Activity Questionnaire
MOS	Medical Outcomes Study Social Support Survey
AUDIT	Alcohol Use Disorders Identification Test
SIQ	Self-Injury Questionnaire
CAST	Cannabis Addiction Test
FTND	Fagerström Test for Nicotine Dependence
ASSIST	Alcohol, Smoking and Substance Involvement Screening Test
DUSI	Drug Use Situations Inventory
ASRS v1.1	Adult ADHD Self-Report Scale
