# Supplementary material for: Psychosocial and Mental Health Determinants of Suicidal Behavior Among Nursing Students: A Cross-Sectional Study in Mexico
Source: Nurs Rep. 2025 Dec 10;15(12):441. doi: 10.3390/nursrep15120441 (PMC12735884; doi:10.3390/nursrep15120441)
Supplement: Supplementary file 1 [file nursrep-15-00441-s001.zip › nursrep-3926283-supplementary.pdf]

nursing reports

MDPI

Supplementary Material

Psychosocial and Mental Health Determinants of Suicidal Behavior Among Nursing Students: A Cross-Sectional Study in Mexico

Margarita L. Martinez-Fierro [1,2], Leticia A. Ramirez-Hernandez [3,*], Perla M. Trejo-Ortiz [1], Georgina Lozano-Razo [4], Javier Zavala-Rayas [4], Sodel Vazquez-Reyes [1], Perla Velasco-Elizondo [1], Alejandro Mauricio-Gonzalez [1], Roxana Araujo-Espino [1], Fabiana E. Mollinedo-Montaño [1], Jose R. Gutierrez-Camacho [1] and Idalia Garza-Veloz [1,*]

[1] Doctorado en Ciencias con Orientación en Medicina Molecular, Unidad Academica de Medicina Humana y Ciencias de la Salud, Universidad Autonoma de Zacatecas, Carretera Zacatecas-Guadalajara Km.6. Ejido la Escondida, Zacatecas 98160, Mexico
[2] Psychology Program, Intercontinental University, Insurgentes Sur Calz. de Tlalpan 4303 Sta Ursula Xitla, Ciudad de México 14420, Mexico
[3] Unidad Academica de Matematicas, Universidad Autonoma de Zacatecas, Zacatecas 98160, Mexico
[4] Unidad Academica de Psicologia, Universidad Autonoma de Zacatecas, Zacatecas 98160, Mexico
* Correspondence: lramirez@uaz.edu.mx (L.A.R.-H.); idaliagv@uaz.edu.mx (I.G.-V.); Tel.: +52-492-925-6690 (ext. 4535) (L.A.R.-H.); +52-492-925-6690 (ext. 4535) (I.G.-V.)

Additional File S1.

Table S1. Reliability and validity of psychological and substance use instruments used in the study.

Instrument	α-Cronbach	Validity	Validation population	Target age Group	Reference
Beck Suicide Ideation Inventory	0.84	Factor loading, subscales: I = 71.2%, II = 62.6%, III = 46.4%	Mexican	$\bar{x}$ = 20.8 years	[37]
Beck Hopelessness Inventory	0.83	Factor loading = 0.4	Mexican	14 to 37 years	[38]
DASS-21	0.86	AVE > 1.0; Total variance explained 46.64%	Mexican	20 to 60 years	[39]
Plutchik Impulsivity Scale	0.713	Kaiser–Meyer–Olkin = 0.814, Bartlett's test of sphericity, Chi^2 = 1883.862 (p < 0.000), variance explained = 47.31%	Mexican and Spaniard	12 to 22 years	[40]
IPAQ	0.8	Factor loading = 0.3	Colombian	18 to 65 years	[41]
ADHD Questionnaire (ASRS V.1.1)	0.88	Total variance explained = 49.26%	Mexican	$\bar{x}$ = 23.34 years	[42]
Social Support (MOS)	0.97	Factor loading = 0.95 to 0.98	Mexican	25 to 80 years	[43]
AUDIT	0.83	Kaiser–Meyer–Olkin and Bartlett's test of sphericity > 0.5 < 0.05, Variance explained > 1.0	Chilean	$\bar{x}$ = 19.2 years	[44]
Self-Injury Scale (CAL)	0.88	Total variance explained = 54.02%.	Peruvian	11 to 18 years	[45]

 https://doi.org/10.3390/nursrep15120441

Instrument	α-Cronbach	Validity	Validation population	Target age Group	Reference
CAST	0.86	Pearson correlation = 0.546	Spanish	16 to 20 years	[46]
FTND	0.86	Total variance explained = 47.7 %	Peruvian	$\bar{x}$ = 20 years	[47]
ASSIST	0.80	Test–retest coefficient of 0.58 - 0.90	Mexican	18 to 23 years	[48]
Drug Use Situations Inventory	0.59–0.92	Test–retest of 0.98, with all items being significant at 0.000.	Mexican	$\bar{x}$ = 26.71 years	[49,50]
RASQ	Internal 0.68 Extra 0.77	Spearman correlation coefficients RASQ internal vs. extra = 0.28 Wald's x^2 = 7.30, p = 0.007; Coef. b = 0.08; e^{B} = 1.08	Caucasian, African, American Latino, Others	19 to 75 years	[51]

DASS-21: Depression Anxiety Stress Scales; IPAQ: International Physical Activity Questionnaire; ASRS V.1.1: Adult ADHD Self-Report Scale Version 1.1; MOS: Medical Outcomes Study Social Support Survey; AUDIT: Alcohol Use Disorders Identification Test; CAST: Cannabis Abuse Screening Test; FTND: Fagerström Test for Nicotine Dependence; ASSIST: Alcohol, Smoking and Substance Involvement Screening Test; CAL: Self-Injury Scale; RASQ: Reasons for Attempting Suicide Questionnaire; AVE: Average Variance Extracted; OR: Odds Ratio; CI: Confidence Interval.

References for this section

36. Macip, S.G.; Martínez, A.D.; León, S.O.; Forteza, C.F.G.; Núñez, J.L.G.J.S.M. Características psicométricas de la Escala de Ideación Suicida de Beck (ISB) en estudiantes universitarios de la ciudad de México. **2000**, *23*, 21-30.
37. Hermosillo-de-la-Torre, A.E.; Méndez-Sánchez, C.; González-Betanzos, F. Evidencias de validez factorial de la Escala de desesperanza de Beck en español con muestras clínicas y no clínicas. *Acta Colombiana de Psicología* **2020**, *23*, 148-169, doi:10.14718/ACP.2020.23.2.7.
38. Gloria Margarita, G.; Patricia, B.; Bonilla, M.; Virseda, J.A. Estrucura factorial y consistencia interna de la Escala de Ansiedad, Depresión y Estrés (DASS-21) en una muestra no clínica. *Psicología y Ciencia Social* **2006**, *8*, 3-7.
39. Alcázar-Córcoles, M.; Verdejo, A.; Bouso, J.C. Psychometric Properties of Plutchik's Impulsivity Scale in Juvenile Spanishspeaking population. *Actas españolas de psiquiatría* **2015**, *43*, 161-169.
40. Mantilla Toloza, S.C.; Gómez-Conesa, A. El Cuestionario Internacional de Actividad Física. Un instrumento adecuado en el seguimiento de la actividad física poblacional. *Revista Iberoamericana de Fisioterapia y Kinesiología* **2007**, *10*, 48-52, doi:10.1016/S1138-6045(07)73665-1.
41. Reyes Zamorano, E.; Godinez, E.; Vargas, K.; Orozco, N.; Medina, J.; Flores, A.; Flores, L.; Jaimes, A.; Ortiz, S.; Lopez, O.; et al. Validación de constructo de la escala de autorreporte del TDAH. *Salud Mental* **2009**, *32*, 343-350.
42. Navarrete, B.; Galindo Vázquez, O.; Alcaraz, R.; Penedo, F.; Lerma, A. Propiedades psicométricas del Cuestionario MOS de Apoyo Social en una muestra de pacientes con enfermedades cardiovasculares en población mexicana. *Psicología y Salud* **2021**, *31*, 225-235, doi:10.25009/pys.v31i2.2691.
43. Seguel Palma, F.; Santander Manríquez, G.; Alexandre Barriga, O. Validez y confiabilidad del test de identificación de los trastornos debidos al consumo de alcohol (AUDIT) en estudiantes de una universidad chilena. J Ciencia y enfermería. **2013**, *19*, 23-35.
44. Jaquelin Kory Cano Quevedo, E.E.G.-G., Roció Yrene Torres Prado, Nancy Elena Cuenca Robles. Adaptación de la Cédula de Autolesión (CAL): Propiedades psicométricas y resultados en una muestra de adolescentes. *Archivos Venezolanos de Farmacología y Terapéutica* **2021**, *4*, 545-549.

45. Fernandez-Artamendi, S.; Fernandez-Hermida, J.R.; Garcia-Cueto, E.; Secades-Villa, R.; Garcia-Fernandez, G.; Barrial-Barben, S. [Spanish adaptation and validation of The Adolescent-Cannabis Problems Questionnaire (CPQ-A)]. *Adicciones* **2012**, *24*, 41-49, doi:10.20882/adicciones.116.
46. Arias-Gallegos, W.L.; Huamani-Cahua, J.C.; Choque-Vera, R. Análisis psicométrico del test de Fagerström de dependencia a la nicotina en una muestra de estudiantes universitarios de Arequipa, Perú %J Acta Médica Peruana. **2018**, *35*, 174-179.
47. Tiburcio Sainz, M.; Rosete-Mohedano, M.G.; Natera Rey, G.; Martínez Vélez, N.A.; Carreño García, S.; Pérez Cisneros, D. Validity and Reliability of the Alcohol, Smoking, and Substance Involvement Screening Test (ASSIST) in University Students. *Adicciones* **2016**, *28*, 19-27, doi:10.20882/adicciones.786.
48. Leon Bello, L.d.J.d. Adaptacion, validacion y confiabilizacion del Inventory of Drug Taking Situation (IDTS) y el Drug Taking Confidence Questionnaire (DTCQ). (Tesis de Licenciatura). Universidad Nacional Autónoma de México, México. Recuperado de https://repositorio.unam.mx/contenidos/397483. **2001**.
49. Torres, L.; Mares, M.; Chainé, S.; VÁZquez, J.; Ruiz, M. *Programa De Satisfactores Cotidianos Para Usuarios Con Dependencia a Sustancias Adictivas. Manual Del Terapeuta*; 2008.
50. Horon, R.; McManus, T.; Schmollinger, J.; Barr, T.; Jimenez, M. A study of the use and interpretation of standardized suicide risk assessment: measures within a psychiatrically hospitalized correctional population. *Suicide & life-threatening behavior* **2013**, *43*, 17-38, doi:10.1111/j.1943-278X.2012.00124.x.

Complementary information: Multivariate data modeling.

We fitted five theory-guided sequential logistic models using complete-case data to identify and weigh risk factors associated with suicidal behavior (dependent variable: dichotomized Beck Suicide Scale, indicating suicidal ideation and/or intent). Each block corresponded to conceptually coherent sets of predictors, and model comparison relied on AIC and McFadden's pseudo-R^2. Collinearity was assessed in every block (all VIF < 5). Continuous predictors (e.g., DASS-21 subscales, hopelessness) were analyzed on their original scales. Higher scores indicate greater symptom severity. The Beck Suicide Scale was dichotomized as 1 to represent ideation/intent, and 0 otherwise. Social support subscales were entered as continuous scores. Analyses used complete-case data. Multivariable modeling added value beyond bivariate tests by simultaneously adjusting for correlated covariates, quantifying independent associations, and comparing parsimony across theory-guided blocks. Signals observed in bivariate analyses (e.g., hopelessness) remained robust after adjustment, while others attenuated, clarifying the relative contribution of each domain.

Model 1 included thirteen sociodemographic, psychosocial, and clinical variables (biological sex, sexual orientation, from another town, children, tobacco use, alcohol use, impulsivity, ADHD screening, hopelessness, DIC emotional support, DIC material help, DIC social leisure/distraction, DIC affective support). Hopelessness showed a strong association with suicidal behavior ($OR = 13.17$; 95% CI: $2.34 - 74.27$; $p = 0.003$). Emotional support appeared protective ($OR = 0.019$; 95% CI: $0.000 - 0.854$; $p = 0.041$), whereas affective support showed an unexpectedly large positive association ($OR = 34.13$; 95% CI: $1.05 - 1108.68$; $p = 0.047$). However, the very wide confidence intervals indicate overfitting and instability given the small sample.

Model 2 comprised eleven clinical and demographic variables (age, biological sex, sexual orientation, socioeconomic status, anhedonia, impulsivity, ADHD screening, DASS-21 stress, DASS-21 depression, DASS-21 anxiety, severe self-harm [more than once]). Depression emerged as the only significant factor, nearly tripling the odds of suicidal behavior ($OR = 2.99$; 95% CI: $1.00 - 8.98$; $p = 0.050$). Anxiety, stress, impulsivity, and socioeconomic status were not significant in this configuration.

Model 3 focused on eight predictors combining social and clinical constructs (sexual orientation, socioeconomic status, impulsivity, DASS-21 depression, hopelessness, severe self-harm, DIC emotional support, DIC affective support). Both socioeconomic status and hopelessness reached statistical significance: lower socioeconomic status was associated with markedly increased risk ($OR = 20.50$; 95% CI: $1.87 - 224.47$; $p = 0.013$), and hopelessness remained significant (OR = 5.95; 95% CI: 1.21–29.14; p = 0.028). Depression remained positive but did not reach significance (p = 0.102); impulsivity was not significant.

Model 4 intentionally reduced the specification to five predictors to improve parsimony and stability -hopelessness, depression, socioeconomic status, and emotional support (impulsivity was not included in this block). Hopelessness ($OR = 2.20$; 95% CI: $1.30 - 3.71$; $p = 0.003$) and depression ($OR = 1.83$; 95% CI: $1.15 - 2.91$; $p = 0.011$) were statistically significant. Socioeconomic status and emotional support preserved their expected directions but did not reach significance.

Model 5 retained only hopelessness and depression. This parsimonious model was the most robust, with narrower confidence intervals and highly significant effects for both predictors: each unit increase in hopelessness doubled the odds of suicidal behavior ($OR = 2.24$; 95% CI: $1.43 - 3.50$; $p < 0.001$), and each unit increase in depression increased the odds by ~54% ($OR = 1.54$; 95% CI: $1.20 - 1.97$; $p < 0.001$).

A concise table of sequential models with adjusted ORs (95% CIs), p-values, complete-case N, and fit indices (AIC, pseudo-R^2) to assist in interpretation (Table S2). These revisions clarify the extra value of the multivariable analysis: most salient bivariate signals—hopelessness and depression—go on after adjustment, while the final parsimonious model (Model 5) captures their independent contributions with stable effect sizes and improved interpretability.

Table S2. Multivariate models with adjusted odds ratios.

Model	Complete cases (n)	AIC	McFadden's pseudo-R^2	Key predictors (adjusted OR [95% CI], p)
**1—Broad socio-psycho-clinical block**	67	100.41	0.188	**Biological sex** 0.11 [0.02 – 0.66], $p = 0.016$; all others ns
**2—Clinical/demographic block**	59	85.56	0.228	**DASS-21 Depression** 3.00 [1.00 – 8.98], $p = 0.050$; all others ns
**3—Focused social/clinical block**	43	49.30	0.475	**Socioeconomic status** 20.50 [1.87 – 224.48], $p = 0.013$; **Hopelessness** 5.95 [1.21 – 29.14], $p = 0.028$; Depression ns
**4—Reduced model (no impulsivity)**	155	153.83	0.156	**Hopelessness** 2.21 [1.31 – 3.74], $p = 0.003$; **DASS-21 Depression** 1.89 [1.19 – 2.99], $p = 0.007$; SES ns; Emotional support ns
**5—Parsimonious (2 predictors)**	294	221.66	0.155	**Hopelessness** 2.24 [1.43 – 3.50], $p < 0.001$; **DASS-21 Depression** 1.54 [1.20 – 1.97], $p = 0.001$
